# Metabolic syndrome and related risk factors among adults in the northern West Bank, a cross-sectional study

**DOI:** 10.1093/inthealth/ihz093

**Published:** 2019-11-01

**Authors:** Basma Damiri, Luna Badran, Deya Safadi, Ahmad Sawalha, Younis Yasin, Mahmoud Sawalha, Moath Amir

**Affiliations:** 1 Faculty of Medicine and Health Sciences, Drugs and Toxicology Division, An-Najah National University, Nablus, Palestine, 00970. P.O. Box 7; 2 Faculty of Medicine and Health Sciences, Department of Medicine, An-Najah National University, Nablus, Palestine, 00970. P.O. Box 7; 3 Department of Medical Laboratory, Thabet-Thabet Hospital, Palestinian Ministry of Health, Ramallah, Palestine, 00970. P.O. Box 1938

**Keywords:** cardiovascular diseases, diabetes, dyslipidaemia, hypertension, International Diabetes Federation, metabolic syndrome

## Abstract

**Background:**

Metabolic syndrome (MetS) is a cluster of cardiometabolic risk factors that includes central obesity, insulin resistance, dyslipidaemia and hypertension. The aim of this study was to establish the prevalence of MetS and its associated risk factors among adult Palestinians using the International Diabetes Federation definition.

**Methods:**

A total of 1348 subjects ages 18–65 y were recruited in a cross-sectional study that was conducted in 2018–2019 in the northern West Bank.

**Results:**

A total of 1082 subjects participated; 51.7% were men. The prevalence of MetS was high (44.9%), and higher among women (46.1%) than men (44.2%) (p<0.001). The prevalence increased significantly with increasing age and body mass index (BMI) in both genders (p<0.001). However, metabolically obese but normal weight individuals (MONW) (8.4%) were also identified, with a slight increase among women (9.4%) compared with men (7.5%) (p=0.56). MetS was more likely to be prevalent among participants with increased fasting blood sugar (5.8 times), increased triglyceride (7.4 times), increased blood pressure (4.5 times) and BMI ≥25 (19.9 times) (p<0.001). The prevalence of MetS was higher among rural (50.3%) vs urban (39.3%) residents and refugees (33.8%).

**Conclusions:**

With increasing age and obesity, clustering of MetS components increased remarkably in both genders. Effective prevention and treatment strategies for MetS and its risk factors should be developed targeting different ages and genders.

## Introduction

Non-communicable diseases, including cardiovascular disease (CVD) and diabetes mellitus (DM), have become the leading causes of mortality and morbidity among Palestinians. They have resulted in substantial direct morbidity and mortality in the occupied Palestinian terrotory.^1^ The prevalence of diabetes was estimated to be high (15.3%) among Palestinians compared with the worldwide prevalence (6%).^2^ Cardiac disease was reported to be the number one cause of death in the occupied Palestinian territory, accounting for 21.0% of all deaths. Hypertension was ranked eighth, accounting for about 5% of all deaths.^3^ Metabolic syndrome (MetS) is a cluster of cardiometabolic risk factors that includes obesity, insulin resistance, dyslipidaemia and hypertension.^4,5^ It has been demonstrated as a common precursor in the development of DM and CVD.^2^ Individuals with MetS are associated with approximately five and twofold increased risk for DM and CVD, respectively.^6^ Therefore MetS is increasingly becoming a challenging public health issue in Palestine.^1^

Few studies have been conducted to establish the prevalence of MetS among Palestinians. These studies have focused on vulnerable groups such as schizophrenia patients,^7^ type 2 diabetes patients in the Gaza Strip,^8,9^ clinic patients in Gaza,^10^ refugee women^11^ and overweight and obese age-targeted groups in the West Bank.^12–14^ Specifically, few studies have determined the general prevalence of MetS among Palestinians in the West Bank and East Jerusalem,^15,16^ and in the Gaza Strip.^17,18^ Moreover, different criteria to diagnose MetS were used, which makes comparisons between studies and tracking changes in MetS prevalence and its abnormalities difficult. A comprehensive understanding of MetS in the adult population may be important for specific direction of prevention strategies. Hypertension, DM and dyslipidaemia are the main risk factors for CVDs in Palestine. These are modifiable factors; therefore, tracking changes in the prevalence of MetS in a society is important for policymakers in order to determine the impact of these changes and provide insights into the extent of the MetS burden in Palestine and explore new risk factors.

This study is a part of ongoing research that aims to establish the prevalence of MetS and to characterize risk factors associated with it among Palestinians in different age groups and different geographical locations. The current study was designed to expand on previous research for a comprehensive understanding of MetS in adult Palestinians. To the authors’ knowledge, this is the first study that has aimed to establish the prevalence of MetS and its associated risk factors among adult Palestinians in the northern West Bank, Palestinian Territories.

## Materials and methods

### Study design

A cross-sectional study was conducted in the northern West Bank, Palestinian Territories, in the Tulkarm and Nablus districts from May 2018 to March 2019.

### Population, sample size and sampling technique

Based on information from the Palestinian Central Bureau of Statistics (2017), the West Bank consists of 11 governorates, 6 of them in the northern West Bank, 3 in the middle and 2 in the south.^19^ Tulkarm and Nablus are two of the biggest governorates in the northern West Bank. The northern West Bank has a population of 1 121 239. There are 570 440 residents in Tulkarm and Nablus, which is 50.8% of the population of the north, and 51.9% of them were adults aged 18–70 y (50.9% males and 49.1% females).^19^ The required sample size was 387 men and 387 women.

In order to give adult subjects an equal chance to participate in this study, the study area was stratified into two strata: cities and villages. Each stratum was then divided into five substrata: central, north, east, west and south. To minimize selection bias, a proportional sample size was chosen based on gender, then based on the location in cities or villages, then on each geographical side. Apparently healthy subjects who attended healthcare clinics in each subarea were recruited to participate in this study through media, flyers, social media and public announcements. To make sure that the subjects were healthy, they were interviewed and were asked about their reason for attending healthcare clinics, types of diseases they had, types of medications they were taking regularly or in the last 6 months, if they had any surgery in the last year, if they had healthcare files in the clinic and if they had certain conditions as explained in the exclusion criteria. The research team stayed at least 2 weeks in each clinic, 7 days/week from 8 am to 5 pm, in order to allow a variety of subjects to participate. Once the research team decided that the subject was apparently healthy and within our inclusion criteria, and to minimize selection bias, every third apparently healthy candidate was chosen to participate in the study (N=1384). Candidates who agreed to participate in the study and signed a consent form were interviewed to answer personal, demographic, lifestyle, family history and socio-economic questions. Participants were then invited to give blood samples after fasting for 12 h. Exclusion criteria included the following medical conditions: subjects with Cushing’s syndrome, hypo- or hyperthyroidism, epilepsy, who were taking regular medications other than antidiabetic or antihyperlipidaemia medications or refused to give a blood sample, who participated in the pilot study and pregnant women.

### Diagnostic criteria

The International Diabetes Federation (IDF) criteria were used to diagnose MetS. The diagnostic criteria, anthropometrics and BP measurements, venous blood collection and biochemical analysis and the accuracy and precision assessment of measurement tools were used and published in previous works.^12–14^ Briefly, to be diagnosed with MetS according to IDF criteria, the individual must have central obesity (defined as a waist circumference ≥94 cm in men, ≥80 cm in women for Caucasians and with ethnicity-specific values for other groups) and at least two of the following: elevated fasting blood sugar (FBS) ≥100 mg/dL or previously diagnosed type 2 diabetes, elevated BP (systolic BP ≥130 mmHg or diastolic BP ≥85 mmHg or treatment of previously diagnosed hypertension), elevated serum triglycerides ≥150 mg/dL or specific treatment for lipid abnormality and reduced high-density lipoprotein (HDL) cholesterol <40 mg/dL in men and <50 mg/dL in women or specific treatment for this lipid abnormality. Blood analysis was carried out at the An-Najah National Hospital laboratories. Blood samples were collected and analysed for blood sugar, triglycerides and HDL using the Cobas C501 Chemistry Analyzer (Roche, Basel, Switzerland) using alfa test kits (Alfa Wassermann, Lisselstein, The Netherlands). The accuracy and precision of anthropometric tools (measuring tapes and scales) and the questionnaire were assessed.

### Data analysis

SPSS version 22 (IBM, Armonk, NY, USA) was used for data entry and analysis. Differences in the means between groups were assessed using the independent samples t test and analysis of variance (ANOVA), whereas Pearson’s χ^2^ or Fisher’s exact test was used for categorical variables. A multivariate binary logistic regression analysis was conducted to evaluate the relative risk by generating the odds ratios (ORs) and 95% confidence intervals (CIs) for metabolic abnormalities and other risk factors. A p-value <0.05 was considered statistically significant.

### Ethics

Ethical approval was obtained from the Institutional Review Board at An-Najah National University in Palestine prior to the research. The study was carried out in accordance with the ethical standards of the Declaration of Helsinki. Consent was obtained from each participant prior to participation. All participants were assured that all data would be confidential and available for the researchers only. The blood tests were free of charge.

## Results

### General population information

Over the 10-month study period, 1348 subjects aged 18–65 y were recruited. The response rate was 80.2%; 266 refused to give blood samples and 1082 participated. Of the 1082 participants, 45.3% were 18–30 y of age, 51.7% were men, 56.1% were rural residents, 64.8% were employed at the time of the study and 40.9% were smokers (34.3% men and 9.2% women) (Table 1). A total of 2.8% of participants were underweight, 32.2% were normal weight, 32.2% were overweight and 33.1% were obese. The most prevalent metabolic abnormalities among participants were increased waist circumference (57.7%) followed by low HDL (51.6%), increased BP (45.5%), increased FBS (33.1%) and increased triglycerides (32.2%) (Table 1).

**Table 1:** Sociodemographic characteristics of respondents

Characteristics	n (%)	Characteristics	n (%)
Gender		Metabolic abnormalities	
Men	560 (51.7)	Increased waist circumference	627 (57.7)
Women	526 (48.3)	Low HDL	560 (51.6)
Work		Increased BP	494 (45.5)
Unemployed	382 (35.2)	Increased FBS	360 (33.1)
Employed	524 (48.2)	Increased triglyceride	349 (32.2)
Student	180 (16.6)	BMI	
Locality		Underweight	30 (2.8)
City	400 (36.8)	Normal weight	346 (32.0)
Village	609 (56.1)	Overweight	348 (32.2)
Camp	77 (7.1)	Obese	345 (33.1)
Age group (y)		Tobacco smoking	
18–20	139 (12.1)	Men	351 (34.3)
21–30	361 (33.2)	Women	94 (9.2)
31–40	191 (17.6)	Total	445 (43.5)
41–50	168 (15.5)		
51–65	220 (20.3)		

### Prevalence of metabolic syndrome and its components based on body mass index (BMI)

The prevalence of MetS was 44.9% (46.1% among women and 44.2% among men). The prevalence was 0.1% among underweight (0.4% among women, 0% among men), 2.7% among normal weight (5.3% among men and 6.6% among women), 14.7% among overweight (37% among men and 28.1% among women) and 27.1% among obese (57.7% among men and 64.9% among women) individuals. The prevalence of MetS increased significantly with increasing BMI and age in both genders and in different localities, including cities, villages and camps, with the highest prevalence among obese adults aged 51–65 y (54.4%). However, metabolically obese but normal weight (MONW) individuals were identified (8.4%), with a slight increase among women (9.4%) compared with men (7.5%), with an OR of 0.79 and a 95% CI of 0.37–1.68 (p=0.56). The prevalence of MetS was higher among rural (50.3%) vs urban (39.3%) residents and refugees (33.8%) (Table 2). Among participants with MetS, the most prevalent components of MetS after increased waist circumference (57.6%) were low HDL (48.5%) followed by high BP (45.3%), high FBS (33.0%) and increased triglycerides (32.1%) (Table 2).

**Table 2:** Prevalence of MetS and its components based on BMI, age, gender and locality

Prevalence	Underweight, n (%)	Normal weight, n (%)	Overweight, n (%)	Obese, n (%)	Total, n (%)	p-Value
BMI						
Men	10 (1.8)	173 (31.1)	204 (36.6)	170 (30.5)	557 (51.5)	<0.001
Women	20 (3.8)	173 (30.0)	144 (27.4)	188 (35.8)	525 (48.5)	<0.001
MetS components						
Waist circumference	2 (0.2)	48 (4.4)	232 (21.4)	344 (31.8)	621 (57.6)	<0.001
FBS	5 (0.5)	63 (5.8)	111 (10.3)	178 (16.5)	356 (33.0)	<0.001
Triglycerides	5 (0.5)	54 (5.0)	112 (10.4)	175 (16.2)	346 (32.1)	<0.001
Low HDL	8 (0.7)	139 (12.8)	172 (15.9)	240 (22.2)	523 (48.5)	<0.001
BP	4 (0.4)	76 (7.0)	150 (13.9)	261 (24.1)	488 (45.3)	<0.001
MetS based on age (y)						
18–20	0 (0)	3 (2.2)	7 (5.0)	12 (8.6)	22 (15.8)	<0.001
21–30	0 (0)	7 (1.9)	34 (9.4)	41 (11.4)	82 (22.8)	<0.001
31–40	0 (0)	2 (1.0)	29 (15.2)	51 (26.7)	82 (42.9)	<0.001
41–50	0 (0)	3 (1.8)	32 (19.0)	73 (43.7)	108 (64.3)	<0.001
51–65	1 (0.5)	14 (6.5)	56 (25.8)	118 (54.5)	189 (87.1)	<0.001
Total 18–65	1 (0.1)	29 (2.7)	158 (14.7)	295 (27.1)	483 (44.9)	<0.001
MetS based on gender						
Men	0 (0)	13 (5.3)	91 (37.0)	142 (57.7)	246 (44.2)	<0.001
Women	1 (0.4)	16 (6.6)	68 (28.1)	157 (64.9)	242 (46.1)	<0.001
MetS based in locality						
City	0 (0)	8 (2.0)	49 (12.3)	100 (25.1)	157 (39.3)	<0.001
Village	1 (0.2)	19 (3.1)	104 (17.2)	181 (29.9)	305 (50.3)	<0.001
Camp	0 (0)	2 (2.6)	6 (7.8)	18 (23.4)	26 (33.8)	<0.001

Significant at p<0.05.

### Gender-based differences in MetS components

Although men were less likely to have an increased waist circumference compared with women (OR 0.69 [95% CI 0.54–0.87]), they were more likely to have increased serum triglyceride (OR 1.98 [95% CI 1.53–2.57]) and increased BP (OR 1.47 [95% CI 1.15–1.87]) (Table 3).

**Table 3:** Gender-based differences in MetS components

Components	Men	Women	OR (95% CI)	p-Value
Waist circumference	297 (53.2)	324 (62.2)	0.69 (0.54–0.87)	0.003
FBS	189 (33.9)	167 (32.1)	1.11 (0.86–1.4)	NS
Triglycerides	218 (39.1)	128 (24.6)	1.98 (1.53–2.57)	<0.001
HDL	272 (48.7)	251 (48.2)	1.02 (0.80–1.29)	NS
BP	278 (49.8)	210 (40.3)	1.47 (1.15–1.87)	0.002

Significant at p<0.05.

### Risk factors associated with MetS

MetS was more likely to be prevalent among participants with increased FBS (5.8 times), increased triglyceride (7.4 times), increased BP (4.5 times) and BMI ≥25 (19.9 times) (p<0.001). Compared with the age group 18–20 y, MetS was 5.6 times more prevalent in the age group 51–65 y (p<0.001) and 2.6 times more prevalent in the age group 41–50 y (p=0.02). Although MetS was more prevalent among rural residents (50.3%) than urban residents (39.3%) and refugees (33.8%), there was no association between MetS and locality. Men were less likely to have MetS than women (OR 0.333 [95% CI 0.211–0.525], p=0.000) (Table 4).

**Table 4:** Factors associated with MetS and its components

Factor	Reference groups	OR (95% CI)	p-Value
Increased FBS	Metabolic abnormalities among non-MetS	5.72 (3.55–9.21)	<0.001
Increased triglycerides		7.41 (4.44–12.37)	<0.001
Low HDL		1.034 (0.670–1.594)	NS
Increased BP		4.47 (1.86–10.75)	0.001
Increased systolic BP		1.93 (0.89–4.20)	NS
Increased diastolic BP		3.03 (1.56–5.87)	0.001
BMI ≥25	BMI <25	19.89 (11.0–36.03)	<0.001
Men	Women	0.33 (0.21–0.53)	<0.001
51–65 y	Age group 18–20 y	5.59 (2.37–13.18)	<0.001
41–50 y		2.58 (1.15–5.81)	0.02
31–40 y		2.09 (0.95–4.62)	NS
20–30 y		1.68 (0.79–3.60)	NS
Urban	Rural	0.81(0.51–1.27)	NS
Refugee		0.49 (0.20–1.20)	NS

Significant at p<0.05.

## Discussion

There were several remarkable findings in this study. The overall prevalence of MetS (44.9%) was higher than anticipated based on previous research in the West Bank. In comparison with local studies, the prevalence of MetS in this study was higher than the estimated population prevalence of MetS (37.0%) in a meta-analysis study using World Health Organization (WHO), IDF and National Cholesterol Education Program Adult Treatment Panel III definitions among adult Palestinians.^1^ It was also higher than the prevalence among Palestinians from east of Jerusalem (36.8%) in 2005 using the IDF definition^16^ and Palestinians from the central part of the West Bank (17%) in 1998 using the WHO definition.^15^ Despite the definition used, MetS prevalence has increased in recent decades, indicating that the response of society and the healthcare system to this epidemic is inadequate. The different criteria used to diagnose MetS in previous studies has made comparisons between studies and tracking changes in MetS prevalence and its abnormalities difficult. More follow-up research is recommended.

Obesity and its impact on associated comorbidities is a major public health problem in different societies and in Palestine.^12,20^ In this study, the overall prevalence of overweight and obesity was high (65%) in this population and was strongly associated with increased age. The overall prevalence of MetS among overweight and obese respondents was also high (64.9%) and was higher among obese (84.8%) vs overweight (45.4%) individuals. These results were greater than what was observed previously among overweight and obese Palestinian refugees^12^ and is even higher than what was estimated by the National Health and Nutrition Examination Survey, which estimated the prevalence of MetS to be 5% among subjects of normal weight, 22% in overweight subjects and 60% in obese subjects.^21^ MetS was 20 times more likely to be prevalent among overweight and obese participants than normal-weight participants. This indicates that at least two-thirds of the population is at high risk for developing chronic diseases such as DM and CVDs, the leading causes of death among Palestinians.^22^ However, overweight, obesity and their related diseases are largely preventable.^23^ An age-targeted prevention approach may be effective and impactful in fostering weight loss among Palestinians.

A unique subset of individuals, termed MONW, was identified in this population. Around 8.4% of normal-weight participants displayed a cluster of obesity-related features, with a slight increase among women (9.4%) compared with men (7.5%). Young women are potentially at increased risk for the development of MetS despite their young age and normal BMI.^24^^–^^26^ Although they have a normal BMI, MONW individuals display a cluster of obesity-related features that may predispose them to the development of MetS and thus CVDs.^24^ Except for central obesity, the most frequent metabolic abnormalities for MONW individuals in this study were similar to those of overweight and obese respondents: low HDL followed by increased BP, increased FBS and increased triglycerides. Therefore screening in individuals with a normal BMI is important in preventing DM and CVDs. The main modifiable risk factors—hypertension, DM, dyslipidaemia and obesity—can be targeted for effective comprehensive prevention programmes. These results take on added importance because MONW individuals are frequently undetected and undiagnosed because of their normal BMI and young age. Therefore gender- and age-targeted screening programmes are essential and recommended.

Similar to previous studies, our results demonstrated that clustering of MetS components increased remarkably with increasing age as well as BMI.^12–14,17,25^ All MetS risk factors were more prevalent in older rather than younger adults. The prevalence of MetS increased around sixfold in the age group 51–65 y compared with the age group 18–20 y. This trend was observed in normal weight, overweight and obese adults. It is known that targeting the younger population to prevent MetS before it happens is a better plan than trying to cure it after it happens. Therefore effective early age-targeted prevention and treatment programmes are required. The healthcare system should be redesigned to address these diseases at earlier ages.

Studies on MetS between genders have shown diverse outcomes.^17,26^ In general, women have a higher prevalence than men of MetS in Arab countries.^27^ In Palestine, a study was conducted in the Gaza Strip that revealed different prevalence trends with genders.^18^ However, other studies concluded there was no gender differences in MetS prevalence among Palestinians in both the West Bank and Gaza Strip.^10,12,13,17^ Our results disagree with the results of these studies. Gender-based differences were also observed in MetS components. Women were more likely to have increased central obesity than men, and central obesity is a prerequisite to a diagnosis of MetS using IDF criteria. This could explain the increased prevalence of MetS among women even though men were more likely to have increased triglycerides and BP.

The worldwide prevalence of MetS ranges from <10% to 84%, depending on the region, urban or rural environment, composition (sex, age, race and ethnicity) of the population studied and which definition of the syndrome is used.^28,29^ MetS is a major public health and clinical challenge worldwide in the wake of urbanization, increasing obesity and sedentary life habits. Different regions and localities have different MetS prevalences. Despite the increased prevalence of obesity among rural residents (67.3%), the higher rates of metabolic abnormalities in rural areas were not expected. This may indicate that the effects of a western lifestyle and diet are permeating more into the West Bank, especially rural areas. In the presence of an epidemic of overweight and obesity as well as a sedentary lifestyle, prevention, identification and treatment of MetS have become a major challenge for healthcare professionals in rural areas specifically and the West Bank in general. Areas undergoing rapid socio-economic, demographic and dietary shifts are among the most vulnerable and require early screening. More studies are required in these areas.

### Strength and limitations

This is the first study that was conducted to investigate MetS in non-vulnerable groups. This is also the first study that identified the presence of MONW among Palestinians. The results of this study have important clinical implications for screening adults. The use of central obesity to identify MONW in this population could be an attractive early screening tool, especially in developing countries. This study has a number of limitations. While this is a representative sample of adults in the northern West Bank, it does not necessarily represent the general Palestinian population. However, it gives an idea about the situation of MetS. In light of the higher rates of overweight, obesity, central obesity and MetS in adults in different areas, more research is needed, especially in the southern West Bank. The lack of prior research studies using the same diagnostic criteria was one of the most important constraints to predict the change in MetS prevalence and risk factors in this area.

## Conclusions

The findings of this study provide evidence-based data on the considerable prevalence of MetS among Palestinian adults. MetS increases with increasing age and obesity. The most prevalent components of metabolic abnormalities that were associated with increased MetS were increased FBS, dyslipidaemia and elevated BP. Therefore action should be taken to slow the rise of preventable non-communicable diseases such as obesity, diabetes, hypertension and dyslipidaemia and thus CVDs. Prevention of obesity needs to be given high priority. Moreover, MONW individuals were also identified. They display a cluster of obesity-related features that may predispose them to the development of MetS. Therefore screening in individuals with normal BMI is important in the prevention of diabetes and CVDs. The Ministry of Health should focus efforts on targeting preventive interventions and awareness campaigns for key risk factors for non-communicable diseases. If no action is taken to reduce these diseases, especially in young adults, these diseases could become an increasing burden for the health system.
